# Connectome analysis for pre-operative brain mapping in neurosurgery[Author-notes FN0001]

**DOI:** 10.1080/02688697.2016.1208809

**Published:** 2016-07-22

**Authors:** Michael G. Hart, Stephen J. Price, John Suckling

**Affiliations:** ^a^ Brain Mapping Unit, Department of Psychiatry, University of Cambridge, Sir William Hardy Building, Cambridge, UK; ^b^ Division of Neurosurgery, Department of Clinical Neurosciences, Cambridge Biomedical Campus, Cambridge, UK; ^c^ Brain Mapping Unit, Department of Psychiatry, University of Cambridge, Herchel Smith Building for Brain and Mind Sciences, Cambridge, UK

**Keywords:** Brain mapping, connectome, echo-planar imaging, glioblastoma, magnetic resonance imaging, neurosurgery

## Abstract

**Object:** Brain mapping has entered a new era focusing on complex network connectivity. Central to this is the search for the connectome or the brains ‘wiring diagram’. Graph theory analysis of the connectome allows understanding of the importance of regions to network function, and the consequences of their impairment or excision. Our goal was to apply connectome analysis in patients with brain tumours to characterise overall network topology and individual patterns of connectivity alterations.

**Methods:** Resting-state functional MRI data were acquired using multi-echo, echo planar imaging pre-operatively from five participants each with a right temporal–parietal–occipital glioblastoma. Complex networks analysis was initiated by parcellating the brain into anatomically regions amongst which connections were identified by retaining the most significant correlations between the respective wavelet decomposed time-series.

**Results:** Key characteristics of complex networks described in healthy controls were preserved in these patients, including ubiquitous small world organization. An exponentially truncated power law fit to the degree distribution predicted findings of general network robustness to injury but with a core of hubs exhibiting disproportionate vulnerability. Tumours produced a consistent reduction in local and long-range connectivity with distinct patterns of connection loss depending on lesion location.

**Conclusions:** Connectome analysis is a feasible and novel approach to brain mapping in individual patients with brain tumours. Applications to pre-surgical planning include identifying regions critical to network function that should be preserved and visualising connections at risk from tumour resection. In the future one could use such data to model functional plasticity and recovery of cognitive deficits.

## Introduction

Neurosurgery requires an understanding of functional anatomy in order to make surgery safe and effective. Hence unsurprisingly neurosurgery has made significant contributions to brain mapping using multiple modalities over many years.^1^ ^,^ ^2^ One of the goals of this endeavour is to accurately predict the functional consequences of lesions (either endogenous or surgically induced) both immediately following surgery and in the long-term (that is, accounting for plasticity induced recovery). This ‘virtual brain’ will require incorporation of localization and network-based approaches to neuroanatomy, and, in doing so, will model brain function in a holistic manner. In other words, brain function is considered as a whole and not limited to one region or network. However, this goal has hitherto proven elusive. Solving the joint problems of modelling brain function predictively would allow one to better plan surgery with regards to timing, extent of resection, and expected recovery.

The development of the connectome, or ‘wiring diagram’ of the brain, offers the potential to answer these questions.^3–7^ Here the brain is viewed as a collection of nodes that are connected via edges.^8^ Connectome analysis has revealed the brain – where nodes are circumscribed brain regions and edges the degree of synchronization of endogenous signals (also known as functional connectivity) – to be organized as a ‘small world’ whereby it parsimoniously balances local specialization with distributed connectivity and short-cuts between regions.^9–11^ In this manner, the brain network shares its small world properties (and others) with a wide variety of other complex networks including social networks, transportation routes, and the world wide web.^12^ The importance of the connectome paradigm in neuroscience research is epitomized by the $40 million Human Connectome Project, which the National Institutes of Health (NIH) has identified as one of the three ‘grand challenges’ for neuroscience research.^13^

Connectome analysis is attractive to neurosurgeons not only for the principle of mapping brain connectivity but also for allowing intuitive modelling of lesions and plasticity. For example, one can remove parts of a network (for example, friends or friendships in a social network) and identify changes in network properties (such as social cohesiveness or rumour propagation) to gain an understanding of the effects at both a local and global level. One can then investigate mechanisms of putative plasticity using models such as connection re-wiring, alternative routes for information flow, or re-activation of redundant pathways.^14^ ^,^ ^15^

To begin to understand the applicability of connectome analysis to neurosurgery, and its potential to answer useful clinical questions concerning functional brain mapping, MRI data depicting blood oxygenation level dependent (BOLD) contrast were acquired pre-operatively from patients with a brain tumour. Using this dataset connectome analysis was undertaken with the following objectives:

1. Derive the connectomes of individual patients with brain tumours.
2. Measure the key features of the connectome and how they compare with those previously described in healthy volunteers.
3. Visualize the connectome in an intuitive and surgically relevant manner.
4. Appraise the applicability of connectome analysis to pre-surgical planning.

## Materials and methods

### Participants

The study was approved by the Local Regional Ethics Committee (protocol number NIHR/CS/009/011) and all participants provided written informed consent. Basic demographic information is summarized in Table 1. All participants had a confirmed glioblastoma at local histological review according to WHO criteria,^16^ and all but one had a complete resection of the contrast-enhancing component as confirmed on a post-operative contrast-enhanced MRI within 72 h of surgery.^17^

**Table 1.** Demographic information.

Patient	Age	Pre-operative examination	Pathology	Operation	Tumour location	Tumour volume (mm^3^)
1	64	Left pronator drift	Glioblastoma	Complete resection	Right superior parietal lobule	35,736
2	73	Intact	Glioblastoma	Complete resection	Right inferior parietal lobule to occipital pole	86,296
3	79	Hemianopia	Glioblastoma	Complete resection	Right inferior occipital lobe	46,232
4	76	Left hemiparesis	Glioblastoma	Biopsy	Right superior para-central lobule	59,304
5	36	Left hemiparesis	Glioblastoma	Complete resection	Right post-central gyrus and supramarginal gyrus	51,544

### Imaging parameters

MRI data were acquired using a Siemens Trio 3T scanner and 16-channel receive-only head coil (Siemens Medical Solutions, Malvern, PA). A multi-echo echo planar imaging sequence continuously acquired BOLD-sensitive data over a period of 10 min and 51 s with a repetition time (TR) of 2.42 s for each three-dimensional volume, resulting in 269 three-dimensional volumes covering the cerebral cortices and cerebellum. Acquisition parameters for resting-state fMRI were the following: flip angle 90°; matrix size 64 × 64; in-plane resolution 3.75 mm; echo times (TE) of 13.00 ms, 30.55 ms, and 48.10 ms; slice thickness 3.8 mm. Anatomical images were acquired using a T1-weighted magnetization prepared rapid gradient echo (MPRAGE) sequence (FOV 256 mm × 240 mm × 176 mm; matrix 256 × 240 × 176; voxel size 1 mm isotropic; TR 2300 ms; TE 2.98 ms; flip angle 9°).

### MRI pre-processing

Connectome analysis of resting-state fMRI offers theoretical advantages over task-based fMRI in accounting for BOLD signal artefacts related to brain tumours. Whereas task-based fMRI is known to be vulnerable to tumour-related susceptibility artefacts, resting-state fMRI data allows removal of noise-related signal in a data-driven and physically principled manner using ME-ICA. Another advantage of resting-state fMRI is that it relies on correlations between time series in the absence of stimulation, whereas task-based fMRI can be susceptible to alterations in neurovascular coupling during task performance.

Data pre-processing was performed using AFNI^18^ (http://afni.nimh.nih.gov/afni/) with custom multi-echo-independent component analysis (ME-ICA) scripts^19^ ^,^ ^20^ to identify and remove non-physiological noise from the BOLD signal. The first 15 s of time points were discarded to allow for magnetization to reach steady state. Subsequent steps included the following: slice time correction, rigid-body motion correction, de-spiking, and de-obliquing. No spatial smoothing or bandpass filtering was performed at this stage.

MPRAGE structural scans were pre-processed with intensity normalization and brain extraction. Standard algorithms for brain extraction resulted in either significant residual non-brain tissue or removal of intra-axial tissue. Therefore, we used the brain atlas of the Montreal Neurological Institute (MNI) defined in a stereotactic coordinate system transformed back into the acquisition spaces of each individual scan to mask the parenchyma of the brain, in a manner similar to that previously reported, but with linear instead of non-linear registration, and the addition of masks of the contrast-enhancing tumour volume that was excluded from the registration cost function.^21^ This allowed robust and automated brain extraction. All images were registered to the Montreal Neurological Institute brain atlas at 2 mm resolution using the FSL package (http://fsl.fmrib.ox.ac.uk/fsl/fslwiki/) FLIRT.^22^ ^,^ ^23^

### Parcellation

**Figure 1.**
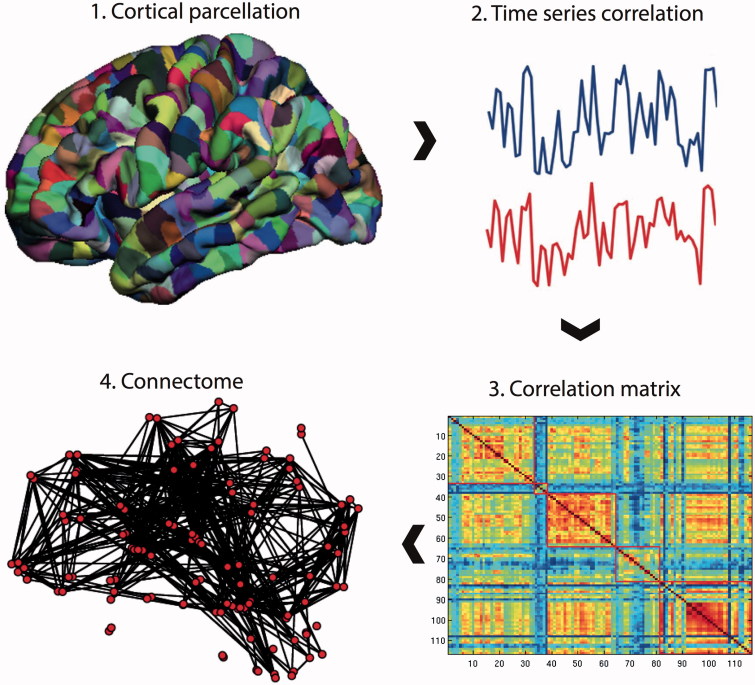
Connectome construction. Methods for performing a connectome analysis using resting-state fMRI data as an example, but similar methods can be applied to data acquired from DTI or EEG/MEG. Initially, a template is chosen to divide the brain into different regions (known as parcels) that form the network nodes. These nodes are used to form the rows and columns of a matrix. Entries of the matrix represent edges between each of the nodes and are formed by recording a measure of statistical dependency (such the Pearson correlation co-efficient) between the resting-state fMRI time series of each node. This correlation matrix can then be thresholded and binarised to form an adjacency matrix, although weighted and fully connected matrices (without thresholding) are also possible. Finally, the co-ordinates of each parcel are used to display the node location onto a surface reconstruction of the brain, with edges representing functional connections.

To form a connectome (Figure 1), pre-processed resting-state fMRI data were parcellated into an anatomical template of 116 regions^24^ (45 regions for each cerebral hemisphere and 26 for the cerebellum). BOLD time series were averaged over the extent of the parcel, and constituted a node in the subsequent network representation.

### Wavelet filtering

A wavelet-based decomposition of the time series was performed to account for frequency-dependent heterogeneity in brain connectivity.^10^ ^,^ ^25^ ^,^ ^26^ Wavelet correlation matrices were formed by applying the maximal overlap discrete wavelet transform (MODWT) to the time series from each parcel. This resulted in a set of five wavelet scales (i.e. frequency bands, scale 1 = 0.2–0.1 Hz, scale 2 = 0.1–0.05 Hz, scale 3 = 0.05–0.03 Hz, scale 4 = 0.03–0.01 Hz, and scale 5 = 0.01–0.006 Hz), at each of which, Pearson’s correlation, *r_ij_*, was calculated between all possible connections between parcels *i* and *j*. Wavelet scales 4 and 5 were not able to produce a matrix of the required mean degree at any threshold and were, therefore, not studied further.

### Thresholding

At each wavelet scale, the network was thresholded; that is, connections were kept if the probability, *p*, of *r_ij_* was greater than the threshold *R*, *p*(*r_ij_*_ _>__ *R*).^10^ As a result, a graphical representation was formed where connections between parcels were either present or absent; in other words, a binary network representation of the connectome. To allow for estimation of small world properties, the mean degree (i.e. mean number of connections associated with a parcel) was chosen to be equal to the log number of parcels: *k*_net_ = 2*log(*n*), following which we defined *R* as the value that resulted in a fixed number of edges = n*k_net_, while controlling for multiple statistical testing by adjusting the probabilistic threshold with the false discovery rate (FDR): *p*(*r_ij_*_ _>__ *R*) < *α*_FDR_ = 0.05. This method of thresholding naturally leads to sparse networks that include only a proportion of all potential connections.^27^

### Graph theory analysis

Network analysis was performed in Matlab [MATLAB 2015a, The MathWorks, Inc., Natick, MA] with the Brain Connectivity Toolbox [http://www.brain-connectivity-toolbox.net] and the R statistical package^28^ with the Brainwaver library (version 1.6).^29^ Specific graph theory measures and their definitions are given in Table 2.

**Table 2.** Definitions of network measures.

Measure	Definition
Centrality	How important a given node’s features are to the overall network
Degree	The number of connections of a node
Efficiency	The inverse of path length (which accounts for disconnected nodes and weights more towards short edges). Can be local (based on a nodes community) or global
Clustering co-efficient, γ	The clustering co-efficient measure of local cliques or nodes whose neighbours are also neighbours of each other and is a measure of network segregation. This can be expressed as a ratio of that from a corresponding random network (‘normalized clustering co-efficient’)
Giant component	Largest connected cluster of nodes in a network
Hub	Nodes that form a key component of the overall network structure e.g. they have high degree, high centrality, or short path length to other nodes
Information centrality	Percentage change in global efficiency due to removal of a single node
Path length, λ	Path length is the number of discrete steps between nodes that are required to complete a journey from one node to another and is a measure of network integration. This can be expressed as a ratio of that from a corresponding random network (‘normalized path length’)
Random error	Removing nodes at random and measuring the change in network properties (e.g. size of the giant component or efficiency)
Resilience	The ability of a network to recovery from removal of specific components (nodes or edges). A network that is highly resilient will demonstrate little change in its graph theory measures after removing node(s) or edges(s). This definition is similar to robustness but implies a dynamic reparative process such as plasticity
Robustness	The ability of a network to maintain its typical graph theory characteristics after removal of specific node(s) or edge(s) i.e. a network that is robust will tend not to change much after removal of specific components
Small world, δ	A measure of simultaneous clustering (network segregation) and short path length (network integration) formed by network short-cuts. Typically defined as γ/λ >1
Targeted attack	Removing nodes based on a ranking of network features (e.g. degree, centrality, or clustering)

Connectome analysis allows the application of measures from graph theory. These include the node degree, defined as the number of connections of each node (i.e. parcel). The clustering co-efficient, a measure of network segregation or local specialization, is defined as the ratio of neighbours of a node that are also neighbours of each other (and, therefore, form a triangle or local clique) over all possible neighbour connections. Path length, which measures network integration or information flow, is the number of steps (or edges) required to move from one node to another. Both the clustering co-efficient and the path length can be compared with the same measures from a randomized network (see below for creation of randomized networks) where they are given the names *γ* and *λ*, respectively. A small world network displays higher clustering but similar path length than a randomized network, and the small world parameter, *δ* = *γ*/*λ*, will be greater than one.

How a network changes after removal of individual node(s) or edges(s) is known as network robustness or resilience. A more robust network is one where removal of a node or edge does not lead to a significant change in graph theory measures (typically the size of the ‘giant’ component, path length, or global efficiency).^10^ ^,^ ^30^ ^,^ ^31^ Robustness can be assessed by either removing nodes in a random manner, or by targeting nodes based on some characteristic such as clustering or path length, known respectively as random error and targeted attack.^32^ Information centrality per node is similar to robustness but defined as the change in global efficiency after removal of a single node.^33^

### Network comparisons

Group analysis was performed to allow robust estimation of overall network measures. This was possible given the overlap in lesion locations and, therefore, in the parcels that were adjacent to the tumour. Group networks were formed by averaging *r_ij_* for each edge at each wavelet scale separately. Network comparisons were based on simulated randomized networks, generated, and configured to match the number of nodes, mean degree, and degree distribution of the brain networks, and simulated scale-free networks of the same number of nodes and edges but with a power law degree distribution. Comparisons between each of these network models were performed with Akaite Information Criteria (AIC).^34^

### Visualization

Brain networks were displayed with the BrainNet viewer^35^ [http://www.nitric.org/projects/bnv/] and Circos^36–38^ on an individual participant basis to highlight the potential application of the analysis to pre-operative brain mapping.

## Results

### Key characteristics of functional brain networks

**Figure 2.**
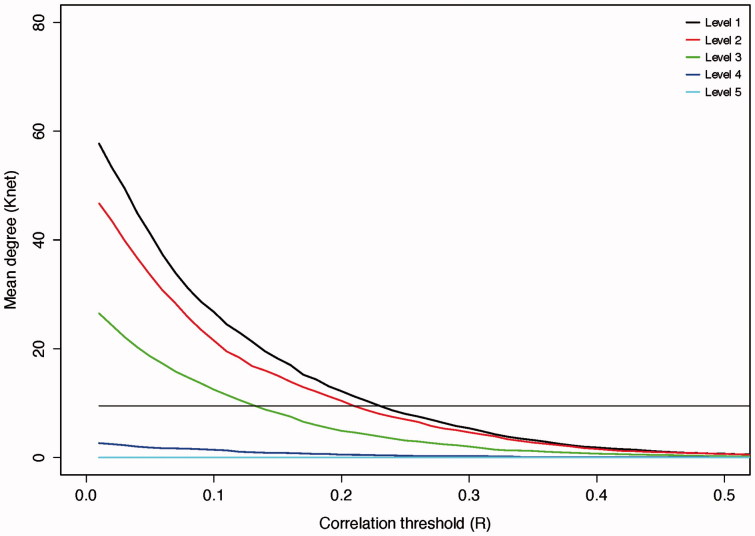
Effects of thresholding on network degree. Increasing the cut-off of the correlation threshold results in a reduction in the number of edges that survive thresholding in the resulting matrix. The straight black line represents the minimum mean degree for small world networks (n*log(*n*) = 9.5). The point of intersection of the wavelet scale degree with this line is used as the threshold to form the binary network used for further analysis. Wavelet scales 4 and 5 were not able to produce a matrix of the required mean degree at any threshold and were, therefore, not studied further.

Small world features were identified over a range of wavelet scales for group average networks (Table 3). Compared with simulated random graphs, brain networks had comparable path lengths but markedly increased clustering, accounting for the associated small world features. Wavelet scale 2 (frequency band 0.5–0.10 Hz) was chosen for further analysis having the highest small world score (*δ* = 1.65, Figure 2).

**Table 3.** Small world features for group networks per wavelet scale.

Scale	Hz	*r*	*R*	*L*_net_	*C*_net_	*λ*	*γ*	*δ*
1	0.10–0.20	0.28	0.23	3.53	0.54	1.50	1.83	1.22
2	0.05–0.10	0.35	0.21	2.72	0.56	1.17	1.94	1.65
3	0.03–0.05	0.41	0.13	2.99	0.61	1.29	1.96	1.51

**Figure 3.**
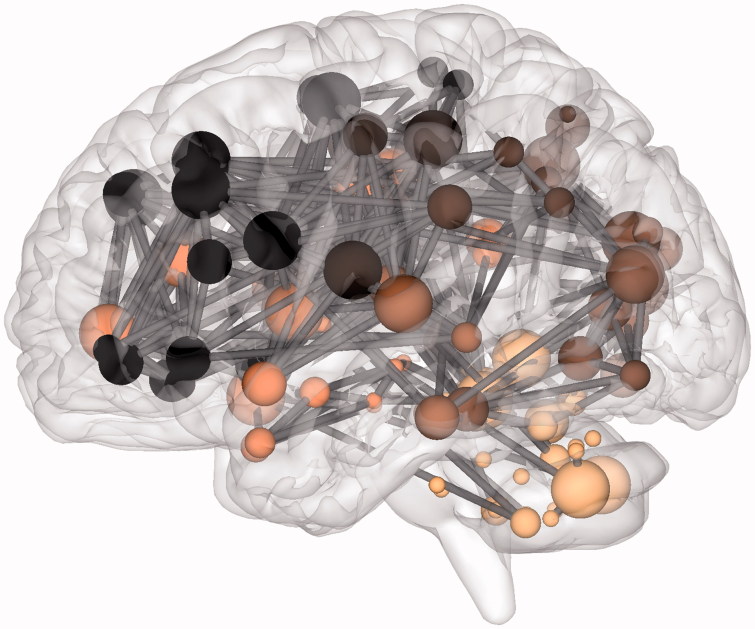
The connectome in glioblastoma. A sagittal view of an individual patient’s connectome at wavelet scale 2. Nodes are coloured according to their anatomical module (e.g. frontal, central, parietal, etc.) and their size is proportional to their degree. Connections (or edges) are presented in grey and represent the binary entries of the adjacency matrix. Locations are based on the co-ordinates of their original parcels and projected onto a surface reconstruction in MNI space.

Increasing wavelet scale represents decreasing frequency. Wavelet scales 4 and 5 are not shown as they did not produce the required mean degree for a small world network (see Figure 3). *r* is the mean correlation of the scale; *R* is the correlation threshold to form the adjacency matrix; *L*_net_ is the mean path length; *C*_net_ is the mean clustering co-efficient; *λ* is the ratio of path length to that of a corresponding random network; *γ* is the ratio of clustering co-efficient to that of a corresponding random network; *δ*, small world measure (*δ* = *γ*/*λ*).

### Anatomical brain networks

The anatomical network for a single participant at wavelet scale 2 is displayed in Figure 3. Thresholding resulted in a sparse group average functional network of 551 edges, or around 8% of all possible edges. While this brain network tended to form a giant component, it did not include all nodes due to the removal of low weight edges during thresholding and the inclusion of small parcels with limited signal-to-noise in their associated time series. Structures outside the giant component included subcortical structures such as the caudate nucleus and putamen which instead tended to form isolated connections to their contralateral homolog. We identified regions of high local connectivity (clustering) in the supplementary motor area and middle cingulate while regions with short path lengths to other regions were found in precuneus and superior frontal gyrus for example (Table 4). Simultaneous local clustering and efficient long distance connectivity (or short cuts between clusters) are the core characteristics of small world organization. Therefore, the connectome effectively carries the brain mapping concepts of functional localization and network connectivity.

**Table 4.** Complete network measures.

Side	Parcel abbreviation	Degree	Clustering	Path length	Information centrality
l	MCIN	31	0.51	2.15	−3.43
l	PQ	31	0.35	1.94	−3.59
r	F1	30	0.41	1.99	−3.26
r	MCIN	28	0.53	2.23	−3.09
r	F2	28	0.51	2.18	−3.10
l	SMA	27	0.62	2.25	−3.01
l	T1	26	0.55	2.45	−2.84
r	T1	26	0.58	2.45	−2.81
r	PQ	25	0.38	2.11	−3.34
l	SMG	24	0.66	2.32	−2.85
l	F2	24	0.46	2.13	−4.44
l	PRE	23	0.68	2.29	−2.82
l	F3OP	23	0.69	2.40	−2.74
r	SMA	23	0.72	2.39	−2.76
r	F3T	22	0.61	2.37	−2.79
l	F1	22	0.49	2.09	−2.96
l	POST	22	0.69	2.25	−2.83
r	POST	22	0.61	2.24	−2.97
r	F3OP	21	0.78	2.38	−2.72
r	SMG	21	0.66	2.32	−2.77
l	IN	20	0.64	2.54	−2.62
l	V1	20	0.46	2.24	−2.86
l	RO	19	0.77	2.59	−2.55
r	PRE	19	0.73	2.30	−2.70
r	F1M	19	0.39	2.29	−3.00
l	O2	19	0.51	2.37	−2.78
l	F3O	18	0.53	2.43	−2.63
l	T2	18	0.56	2.32	−2.74
l	F1M	17	0.38	2.34	−2.80
l	LING	17	0.47	2.24	−3.40
r	RO	15	0.72	2.64	−3.94
l	PCL	15	0.81	2.49	−2.52
l	Q	14	0.67	2.31	−2.59
r	Q	14	0.69	2.52	−2.44
l	O3	14	0.66	2.41	−2.52
r	T2	14	0.44	2.37	−2.87
r	LING	13	0.74	2.49	−2.42
l	P2	13	0.51	2.43	−2.50
r	IN	12	0.62	2.85	−2.29
l	PCIN	12	0.56	2.43	−2.51
r	V1	12	0.82	2.54	−2.38
r	O3	12	0.73	2.64	−2.31
l	P1	11	0.56	2.44	−2.47
r	P2	11	0.64	2.52	−2.41
r	CVCU	10	0.44	2.37	−4.03
l	O1	10	0.53	2.91	−3.51
r	FUSI	10	0.38	2.83	−3.76
r	PCL	10	1.00	2.74	−2.25
l	F3T	9	0.86	2.68	−2.28
l	AG	9	0.64	2.57	−2.32
r	AG	9	0.69	2.61	−2.31
l	T1P	9	0.42	2.89	−2.42
l	ACIN	8	0.46	2.85	−2.21
r	ACIN	7	0.52	2.89	−2.15
r	O2	7	0.86	2.84	−2.08
l	HES	7	0.95	2.97	−2.09
r	CHS	6	0.27	3.46	−2.16
r	F1MO	6	0.67	3.10	−1.95
l	FUSI	6	0.67	2.89	−2.03
r	P1	6	0.67	2.84	−2.06
r	T1P	6	0.40	3.25	−1.94
l	CHCU	6	0.40	3.05	−3.21
l	CHS	6	0.27	2.93	−2.64
r	CVD	5	0.40	3.07	−3.15
r	F3O	5	0.50	3.00	−2.01
r	GR	5	0.30	3.13	−2.21
l	F1MO	4	0.50	3.20	−1.85
r	CAU	4	0.33	1.33	−0.50
r	T2P	4	0.33	3.25	−1.84
r	CHCU	4	0.33	3.13	−3.06
r	F2O	3	0.67	3.94	−1.45
r	PCIN	3	1.00	2.87	−1.98
l	HIP	3	0.67	1.25	−0.20
l	PHIP	3	0.33	1.25	−0.28
r	PHIP	3	0.67	1.25	−0.20
l	F1O	3	0.67	3.94	−1.45
r	F1O	3	0.33	4.05	−1.51
l	PUT	3	0.33	1.67	−0.36
l	THA	3	0.33	1.67	−0.36
l	F2O	3	0.33	3.05	−2.44
l	CHSS	3	0.00	3.83	−2.58
r	CHSS	3	0.00	4.36	−3.17
l	GR	2	0.00	4.09	−1.40
r	HIP	2	1.00	1.75	−0.16
r	O1	2	1.00	3.75	−1.53
l	CAU	2	1.00	1.83	−0.22
r	PUT	2	1.00	1.83	−0.22
l	T2P	2	1.00	3.79	−1.54
r	CHIS	2	0.00	5.32	−1.94
l	CHG	1	−1.00	1.00	−0.06
r	CHG	1	−1.00	6.31	−0.89
l	CHB	1	−1.00	1.00	−0.06
r	CVT	1	−1.00	4.06	−1.34
l	AMYG	1	−1.00	2.00	−0.13
l	PAL	1	−1.00	2.50	−0.17
r	THA	1	−1.00	2.50	−0.17
r	HES	1	−1.00	3.63	−1.57
l	T3	1	−1.00	3.90	−1.44
r	T3	1	−1.00	3.82	−1.46
l	CHIS	1	−1.00	4.82	−1.14
l	CHCL	1	−1.00	4.03	−1.36
r	CHCL	1	−1.00	4.11	−1.32
r	CHB	0	−1.00	−1.00	0.00
l	CHT	0	−1.00	−1.00	0.00
r	CHT	0	−1.00	−1.00	0.00
l	CHF	0	−1.00	−1.00	0.00
r	CHF	0	−1.00	−1.00	0.00
l	CVL	0	−1.00	−1.00	0.00
l	CVCL	0	−1.00	−1.00	0.00
r	CVP	0	−1.00	−1.00	0.00
l	CVU	0	−1.00	−1.00	0.00
l	CVN	0	−1.00	−1.00	0.00
l	OC	0	−1.00	−1.00	0.00
r	OC	0	−1.00	−1.00	0.00
r	AMYG	0	−1.00	−1.00	0.00
r	PAL	0	−1.00	−1.00	0.00

Network measures are shown for all parcels in order of descending degree. Note that for the last parcels where the degree is the lowest the parcel can be outside of the main giant component of the network making clustering and path length values inaccurate. Parcel abbreviations are the same as in Figure 7. Regions are ranked in order of decreasing degree.

### Defining network ‘hubs’

**Figure 4.**
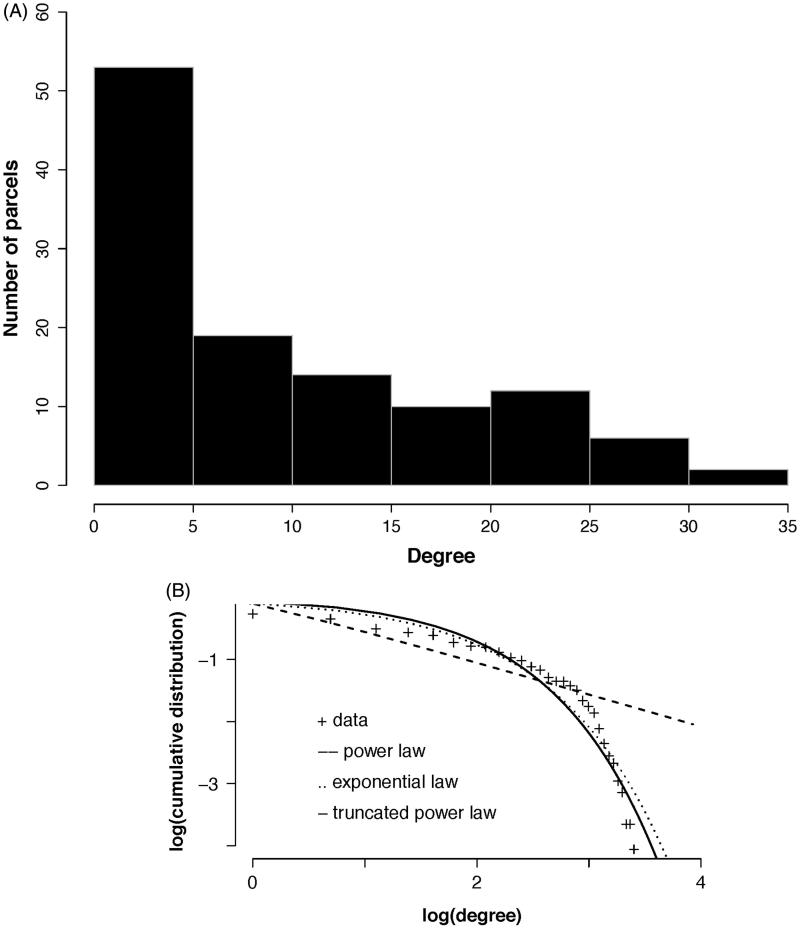
Degree distribution. (A) The histogram for the group network node degrees. The majority of nodes are of low degree (<5) while the maximum degree extends above 30 (although few nodes have this degree). (B) The group network degree distribution is compared to that from simulated networks with either an exponential, power law, or exponentially truncated power law degree distribution. The best fit determined using Akaite Information Criteria was with the exponentially truncated power law degree distribution.

The susceptibility of the brain network to injury is analytically dependent on the degree distribution. The degree distribution of our empirical group averaged functional brain network demonstrated a heavy tailed distribution that best fit an exponentially truncated power law (Figure 4). This degree distribution defines the existence of hubs as those nodes with disproportionately high connectivity, but form a minority of all nodes in the network (Table 4).

### Mapping robustness to injury

**Figure 5.**
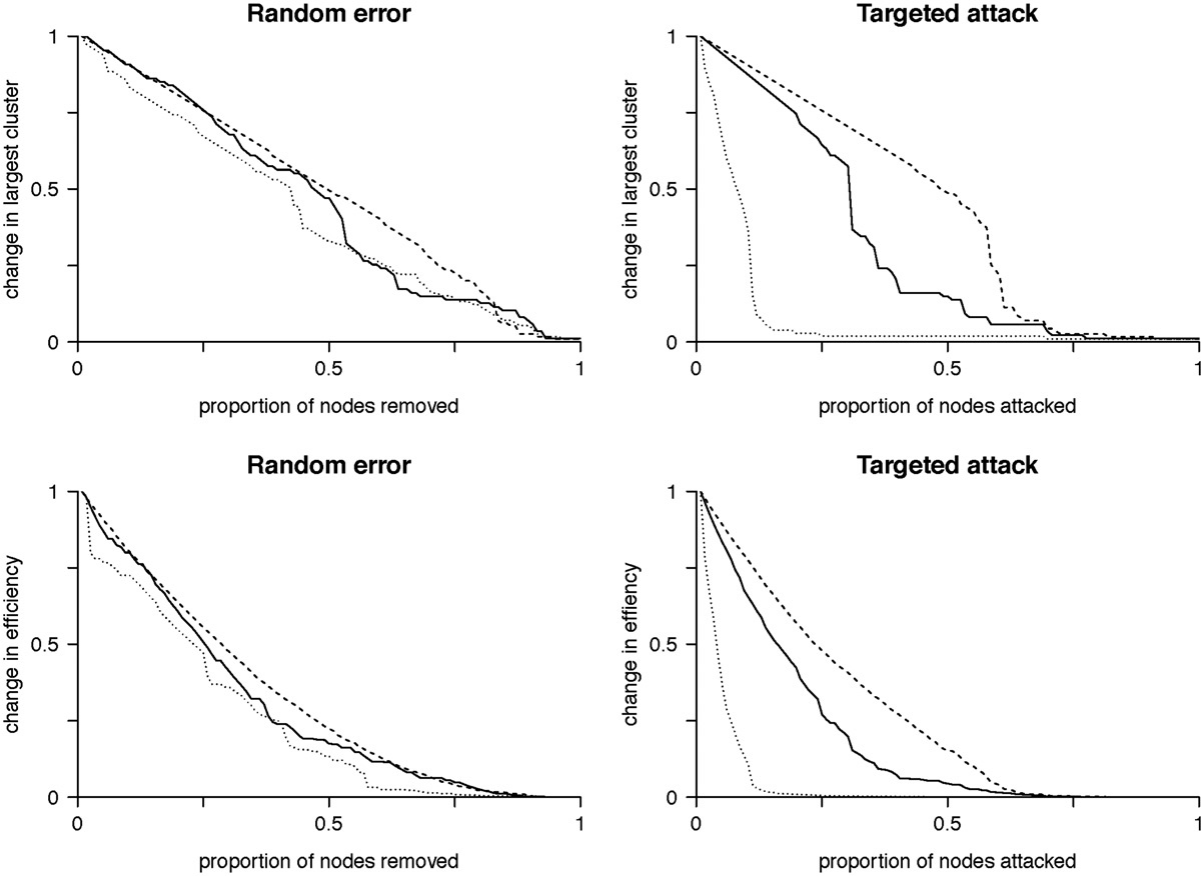
Random error and targeted attack. The change in the size of the network giant component (top row) or efficiency (bottom row) due to either random error (left column) or targeted attack based on degree centrality (right column). Changes are relative to the values for the intact network. All networks are approximately equally affected by random error. However, targeted attack reveals vulnerability of the scale free network, while the brain network is of intermediate vulnerability between the scale-free and random networks. Horizontal axis values are the proportion of nodes removed and vertical axis values are scaled to maximum. Solid line = brain networks, dotted line = simulated scale-free networks, dashed line = simulated random networks

**Figure 6.**
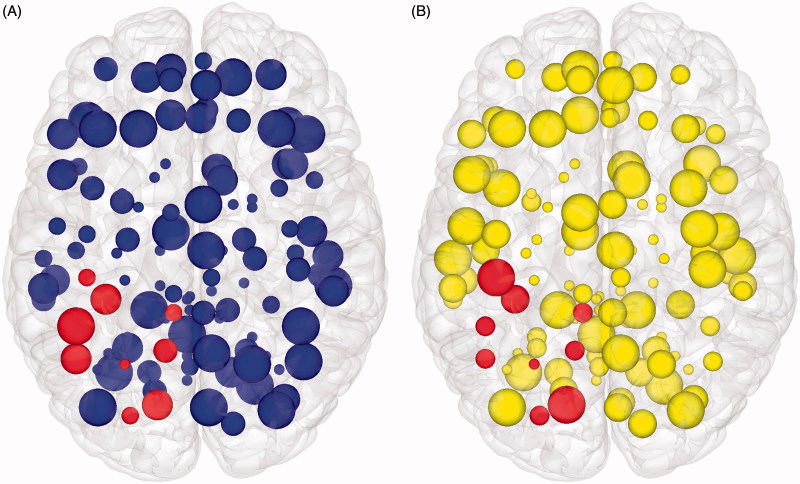
Brain mapping with graph theory network measures. Axial view of node features displayed in cortical surface reconstructions. (A) Node size is proportional to clustering co-efficient. (B) Node size is proportional to information centrality. In both figures, those nodes that are spatially adjacent to the tumour are highlighted. Network edges are removed to focus on the node features. If one were to use this information for pre-surgical planning, purposefully sacrificing selected smaller nodes to allow an extended surgical resection could be seen as having a minimal effect on overall network efficiency (and, therefore, by extrapolation on higher cognitive features such as intelligence). However, inadvertently affecting too many of larger nodes would be expected to have a disproportionate effect on overall network efficiency, and, therefore, should be avoided.

The existence of hubs suggests random removal of nodes will have minimal effect on the overall topology as most nodes are of low connectivity, whereas focused removal of hubs will more likely have a significant effect on topology. Random node removal found our group averaged functional brain network to be as robust as both simulated randomized and scale-free networks (Figure 5). In this manner, the brain is remarkably tolerant to small areas of injury that occur at random. In comparison, targeted attack breaks down the brain network earlier, with the brain network demonstrating intermediate vulnerability between the scale-free and random networks, which is consistent with specific nodes being highly vulnerable to injury and acting as ‘weak links’ in the network. Information centrality can subsequently be generalized to determine the effects on the network of removing each node on overall network efficiency (Table 4). Information centrality identifies a core of highly vulnerable nodes which partially, but not fully, overlap those based on other measures of centrality or are otherwise defined as network hubs, but with more clinically intuitive inference (Figure 6). If one were to use this information for pre-surgical planning, one could purposefully sacrifice selected nodes whose loss would be predicted to have less effect overall network efficiency (and, therefore, by extrapolation on higher cognitive features such as intelligence). Furthermore, nodes that have a disproportionately large role in overall network efficiency could be avoided.

### Network effects of tumours

One can use a network approach to visualise the connectivity that is either lost, or is at risk of being lost due to real (rather than simulated) lesions (Figure 7). Compared with the contra-lateral hemisphere, brain tumours produced clear and consistent effects at an individual subject level including reduced connectivity at intra-lobar, intra-hemispheric, and inter-hemispheric scales. Another perspective to view these data is as the ‘connections at risk’ by removing a specific region adjacent to the tumour, for example, if one wished to include a resection margin around the lesion (Figure 7, red edges). In each participant, the effects of an extended tumour resection were not only local but also included long-range connections both within and between hemispheres. Therefore, to fully understand the effects of surgery on brain function, one must use an approach that considers the connectivity of the brain in its entirety.

Figure 7. Connections at risk. Circular representations of brain functional connectivity data (individual patient data, wavelet scale 2). Images are in neurological projection (image left = left hemisphere) with superior aspect of the image representing anterior brain (akin to an axial view). The sides are symmetrical representations of individual lobes (and parcels within) in their anterior–posterior co-ordinates. Inner circular heatmaps represent degree, clustering, and information centrality (outside to inside) per parcel. Lines representing intra-lobe connections are outside with inter-lobe connections in the centre. On the right, the nodes closest to the tumour are highlighted in red, while on the left the homologous nodes from the contralateral hemisphere are shown for comparison. The tumour was associated with reduced connectivity at intra-lobar, intra-hemispheric and inter-hemispheric levels. These effects were clear and consistent at the individual participant level. If one were to use this for tumour planning, then the connections in red would represent those that could be affected by extending the resection outside of the contrast enhancing margin, and would then become ‘connections at risk’. Parcel codes (alphabetical): ACIN: anterior cingulate; AG: angular gyrus; AMYG: amygdala; CAU: caudate; CHB: biventricular; CHCL: central lobule; CHCU: culmen; CHF: floculus; CHG: gracilis; CHIS: inferior semilunar; CVL: lingual; CHS: simplex; CHSS: superior semilunar; CHT: tonsil; CV: vermis; F1M: superior medial frontal; F1MO: superior frontal medial orbital; F2: middle frontal; F20: middle orbital; F30: inferior frontal pars orbitalis; F3OP: inferior frontal pars opercularis; F3T: inferior frontal pars triangularis; FUSI: fusiform; GR: gyrus recturs; HES: Heschl gyrus; HIP: hippocampus; IFG: inferior frontal gyrus; IN: insula; LING: lingual; MCIN: middle cingulate; O1: inferior occipital; O2: middle occipital; O3: superior occipital; OC: olfactory cortex; P1: superior parietal lobule; P2: inferior parietal lobule; PAL: lentiform nucleus; PCIN: posterior cingulate; PCL: paracentral lobule; PHIP: parahippocampal gyrus; POST: post-central; PRE: precentral; PQ: precuneus; PUT: putamen; Q: cuneus; RO: rolandic operculum; SMA: supplementary motor area; SMG: supramarginal gyrus; T1: superior temporal; T1P: temporal pole; T2: middle temporal; T2P: middle temporal pole; T3: inferior temporal; THA: thalamus; V1: calcarine.

**Figure:**
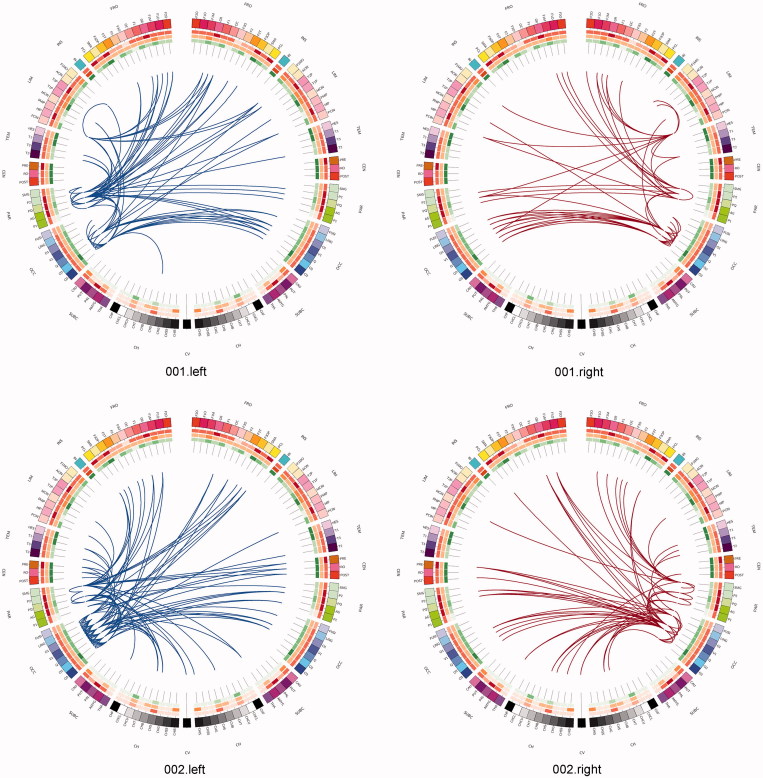

**Figure:**
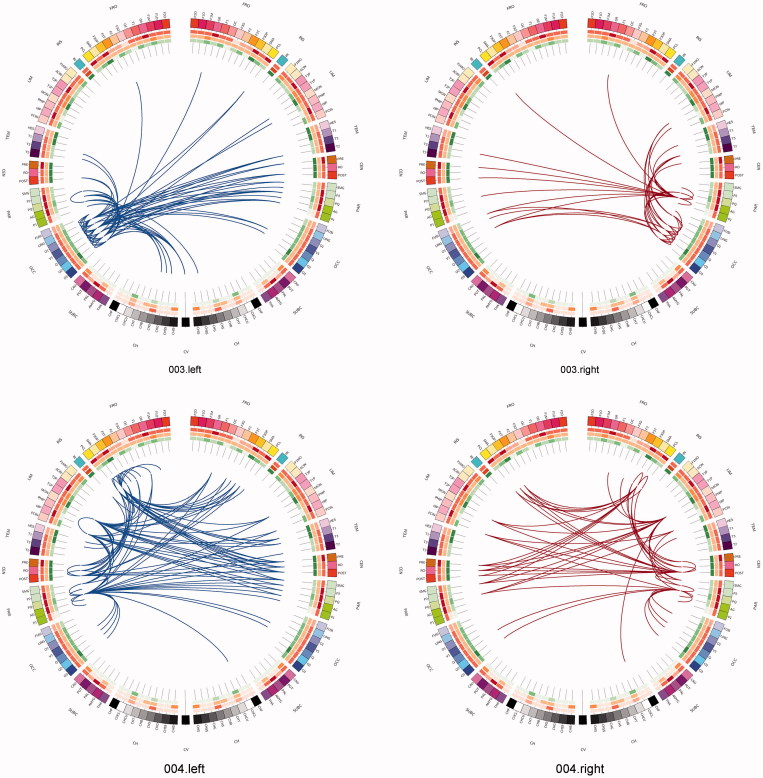

**Figure:**
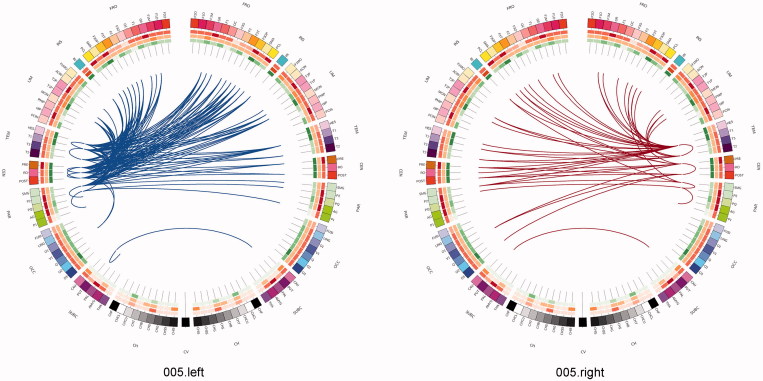

## Discussion

We present the first comprehensive analysis of the functional connectome in patients with brain tumours. Key achievements were validating the methodology behind connectome construction and identifying the core network features that have previously been identified in healthy controls. Functional brain networks were simultaneously locally clustered, but highly efficient with effective short cuts that formed a quintessential small world.^9^ ^,^ ^11^ We also expanded on the technical aspects of network analysis to include measures specifically relevant to neurosurgery, such as information centrality.^33^ Finally, we demonstrated the relevance of network analysis to neurosurgery, including brain mapping of specific network features, understanding the effects of lesions, and visualizing the data in an intuitive yet principled manner.^39^

Mapping hubs (and indeed other graph theory-related measures such as efficiency and path length) creates a new vocabulary to use for functional brain mapping. This can be used for pre-surgical planning to preserve nodes that are critically important to the network composition while tolerating the removal of other nodes that may have less effect on the network. Furthermore, the potential long-range connectivity that is at risk from resection of the tumour can be isolated, characterized, and preserved. This method could be expanded to model virtual lesions on control networks and comparing them with empirical networks to identify putative plasticity. The ability to model real lesions and potential plasticity are key requirements for any future ‘virtual brain’.

Current brain-mapping techniques (e.g. cortical stimulation and task-based functional MRI) are immensely useful, particularly at mapping local function or individual networks,^40–44^ but are usually constrained to identifying a focus of maximal activation for a specific function that is in turn deemed most relevant to the proposed surgical outcome. Theories of functional neuro-anatomy that have been applied to these methods have been those of localization or regional specialization (e.g. primary cortex function) and brain circuit connectivity (e.g. the Wernicke–Geschwind language circuit). Connectome analysis naturally balances these theories of functional localization and network connectivity in a small world framework (and other related concepts)^45^ and can, therefore, be viewed as complimentary to these established models.

One of the key requirements for the connectome to gain acceptance in the neurosurgical domain is to confirm the relationship of graph theory measures to neuro-cognitive outcome.^46^ Connectome analysis is a relatively recent field; therefore, most of the data on neuro-cognitive outcomes is in healthy controls. The efficiency of a network is related to general intelligence quotient,^47^ ^,^ ^48^ while the extent of small world features is negatively correlated with the performance of a task and higher education,^47^ suggesting that the degree of small worldness functions as a rheostat depending on the perceived complexity of the task. Preliminary work on patients with low-grade glioma have found a small worldness and efficiency are related to cognitive deficits and intelligence in participants with frontal lobe tumours.^50^ ^,^ ^51^ Overall it appears that the basic measures of connectome analysis are related to core neuro-cognitive measures in healthy controls and potentially in patients with tumours too although the data is currently somewhat limited. A novel approach to improving the cognitive relevance of connectome analysis would be to use a parcellation based on resting-state state fMRI networks that have been shown to correspond to task-based functional activations in a hierarchical clustering model.^52^ ^,^ ^53^ Therefore, the resulting network would be the interplay of different functional components, allowing more intuitive inference of network links and their pathophysiology.

While our cohort is of sufficient size to demonstrate the core network features and provide a proof of principle with our methods, these data could be improved upon by including a larger cohort with a variety of lesion locations and pathologies. Longitudinal imaging with neuro-cognitive outcome data will aid not only in validating the consistency of network metrics but also in identifying dynamic network changes that could be related to either plasticity or decompensation depending on the direction of cognitive effect.^54^ ^,^ ^55^ Practically this process should be reasonably straightforward and achievable with minimal infrastructural investment. Most modern MRI scanners can acquire resting-state fMRI data, the sequences themselves last less than 10 min, neuro-cognitive assessment is readily being viewed as standard practice for many types of neurosurgery both at presentation and during follow-up.^56^ ^,^ ^57^ Successfully integrating these research protocols into routine clinical practice will be critical to establishing connectome analysis in neurosurgery as well as in developing high-quality datasets.

Another aspect that could be developed further is the technical methodology behind connectome construction and analysis. For constructing the first connectomes in participants with real lesions, we wished to concentrate on the least complicated and most established methods of network construction, such as binary thresholded networks based on anatomical parcellations.^10^ ^,^ ^58^ This was to allow our methods to be valid and understandable yet not to distract from our key message, which was what a connectome analysis could bring to neurosurgery. Now we have established the key features and challenges of connectome analysis in a neurosurgical population, there is now a foundation with which to base further development of our methods. Improvements could be by applying more refined parcellation templates,^59^ ^,^ ^60^ exploring alternative measures of statistical dependency between nodes (such as partial correlation or mutual information),^61^ preserving edge weights, and obviating thresholding to create fully connected graphs.^62^

Finally, the field of network dynamics, or how information flows over the network, is a largely untapped avenue to explore.^63^ ^,^ ^64^ Network approaches (known as cascading failures) have been successfully applied to studying blackout propagation in power networks^65^ and traffic jams.^66^ ^,^ ^67^ Similar approaches could be applied in the brain to study seizure propagation and model the effects of cortical stimulation. This latter approach would view cortical stimulation as local overload leading to a network ‘blackout’, which not only allows the creation of an analytical model but also allows extrapolation of local effects to a network level. To use an analogy with a power grid network, cortical stimulation could be looked upon as overloading certain links, which then results in information overload throughout the network and subsequent functional shut down. Such a solution may resolve the discrepancy in why function appears to be so variable between individuals.^68^ Refinement of this model offers a unique opportunity to pre-operatively predict the likely sites for positive stimulation, thereby marrying old and new techniques of brain mapping to result in improvements in patient care.

## Conclusions

We present the principles underlying a connectome analysis of functional brain data in patients with brain tumours and demonstrate how analytically principled methods can be used to explore the key features of these networks. With these initial results, we hope to have demonstrated the potential of connectome analysis to addressing important questions in functional neuroanatomy. Understanding how the brain copes with and responds to lesions from a network perspective may bring us one step closer to developing a working ‘virtual brain’ to plan surgery in a way that is not only safer but also allows more extensive surgical resection.
